# Neuroendoscopic-assisted versus mini-open craniotomy for hypertensive intracerebral hemorrhage: a retrospective analysis

**DOI:** 10.1186/s12893-022-01642-8

**Published:** 2022-05-14

**Authors:** Wenchao Lu, Hui Wang, Kang Feng, Bangxu He, Dong Jia

**Affiliations:** 1 Department of Neurosurgery, The Xi’an Daxing Hospital, Xi’an, Shaanxi China; 2 Department of Neurosurgery, The Xi’an Fengcheng Hospital, No.9 Fengcheng Third Road, Xi’an Economic and Technological Development Zone, Xi’an, Shaanxi China

**Keywords:** Hypertensive intracerebral hemorrhage, Neuroendoscopy, Mini-open craniotomy, Prognosis

## Abstract

**Objective:**

To compare outcomes in neuroendoscopic-assisted vs mini-open craniotomy for hypertensive intracerebral hemorrhage (HICH), so as to provide reasonable surgical treatment.

**Methods:**

Clinical data of 184 patients with HICH in the hospital from January 2019 to May 2021 were analyzed retrospectively. The patients were divided into mini-open craniotomy group and neuroendoscopic-assisted group. The operation time, hematoma clearance rate, intraoperative blood loss, neurological function recovery, and postoperative mortality of the two groups were compared by retrospective analysis.

**Results:**

The operation time and intraoperative blood loss in the mini-open craniotomy group were more than those in the neuroendoscopic-assisted group, but there was no significant difference between the two groups. There was no significant difference in hematoma clearance rate between the two groups, but for the rugby hematoma, the hematoma clearance rate in the neuroendoscopic-assisted group was higher than in the mini-open craniotomy group, the difference was statistically significant. Within 1 month after the operation, there was no significant difference in mortality between the two groups. 6 months after the operation, there was no significant difference in the recovery of neurological function between the two groups.

**Conclusion:**

Neuroendoscopic-assisted and mini-open craniotomy for the treatment of HICH has the advantages of minimal trauma with good effects, and its main reason for short operation time, reduced bleeding, and high hematoma clearance rate. Although the two surgical methods can improve the survival rate of patients, they do not change the prognosis of patients. Therefore, the choice of surgical methods should be adopted based on the patient's clinical manifestations, hematoma volume, hematoma type, and the experience of the surgeon.

## Introduction

Hypertensive intracerebral hemorrhage (HICH) is a common cerebrovascular disease, which is one of the complications of hypertension. HICH has been reported to account for 50–70% of all spontaneous intracranial hemorrhage (ICH), its morbidity and mortality both occupy the top among all types of strokes, more than 30% of survivors suffer from varying degrees of disability [1, 2]. the incidence of HICH continues to rise with the aged tendency of the population [3]. Because of its high incidence rate, high mortality, and disability, it has become an important public health problem in the world, [1]. At present, the treatment of HICH is mainly divided into conservative treatment and surgical treatment. The purpose of surgical treatment is to effectively reduce the compression of hematoma on the surrounding brain tissue; reduce the intracranial pressure; protect the penumbra brain tissue, to minimize the functional damage caused by cerebral hemorrhage [4]. Mini-open craniotomy and neuroendoscopic-assisted hematoma removal techniques both have the advantages of reduced trauma, fewer complications, and better curative effects [5]. How to choose an appropriate surgical method is closely related to the prognosis of patients. In this study, clinical data of 184 patients with HICH who received treatment in our hospital from January 2019 to May 2021 were analyzed retrospectively, compare outcomes in neuroendoscopic-assisted vs mini-open craniotomy for HICH, to provide reasonable surgical treatment.

## Materials and methods

### Clinical data

Clinical data of 184 patients with HICH who underwent treatment at our hospital from January 2019 to May 2021 were analyzed retrospectively. This study included 101 males and 83 females. The age range was 25–69 years, with an average age of 56 ± 6.2 years old. According to the operation methods, the patients were divided into the mini-open craniotomy group and the neuroendoscopic-assisted group. Before the operation, the blood pressure was controlled at an acceptable level (systolic blood pressure ≤ 140 mmHg, diastolic blood pressure ≤ 90 mmHg).

### Inclusive criteria

(1) Computed tomography (CT) showed basal ganglia hemorrhage with or without ventricular hematoma; (2) History of hypertension; (3) There was no history of head trauma; (4) The surgical indications were clear (cerebral hernia formation, midline deviation > 1 cm) and/or progressive deterioration of consciousness (Glasgow Coma Scale (GCS) decrease ≥ 2 points).

### Exclusive criteria

(1) Bilateral mydriasis, no spontaneous breathing; (2) Hemorrhage of the brain stem or cerebellum; (3) Hemorrhage caused by intracranial aneurysms, arteriovenous malformations, blood system disease, etc. (4) Patients with obvious contraindication of operation or family members refusing surgical treatment.

## The operation mode was as follows:

1. Mini-open craniotomy group: After general anesthesia, the patient was placed in a supine position with the head turned to the opposite side of the hematoma. Based on the CT images, make a skin incision about 5 cm long at the projection of the body surface at the largest level of the hematoma. Routine disinfection of surgical area、drape, craniotomy, skull drilling, the bone flap with a diameter of 2–3 cm was formed by milling cutter, arc cutting of dura. Under the microscope, after the separation of the sulci, a fistula is created(about 1 cm) into the hematoma cavity. the hematoma was slowly aspirated, and look for the bleeding point directly, bipolar electrocoagulation was performed, and the hematoma cavity was repeatedly washed with normal saline to confirm that there was no active bleeding. Dura with tension-reducing suture, and the cranium was reset and the temporal muscle, subcutaneous and scalp were sutured intermittently.
2. Neuroendoscopic-assisted group: Based on the CT images, avoiding the cortex of the functional area, make a 4 cm incision mark on the skin with the extension of the long axis of the hematoma and the intersection of the scalp as the center. Routine disinfection of surgical area、drape, craniotomy, skull drilling, the bone flap with a diameter of 2–3 cm was formed by milling cutter, After cauterizing the dura, the dura was incised and the cortex was electrocoagulated. The puncture sleeve with tube core was used to puncture the cortex, after reaching the hematoma cavity, the tube core was removed, and the neuroendoscope was placed through the sleeve to remove the hematoma under the endoscope, in general, 60–90% of the hematoma can be removed. In case of bleeding, electrocoagulation can be used, and a drainage tube should be placed after no active bleeding, the bone flap was fixed in situ and the scalp was sutured intermittently.

If patients with cerebrospinal fluid circulation obstruction caused by hematoma breaking into the ventricular system, lateral ventricle drainage should be performed before surgery.

### Postoperative treatment

After the operation, the vital signs and the changes in organ function were observed. Keep the respiratory tract unobstructed and prevent complications such as peptic ulcer and respiratory tract infection, routine analgesia and sedation were given, intravenous application of antihypertensive drugs to avoid blood pressure fluctuations. All patients underwent CT scans at 1, 3, 7 and 14 days after the operation to evaluate the changes of hematoma. The patients were followed up for 6 months.

### Efficacy evaluation

The hematoma volume was calculated by Broderick’s method [6] (Length × width × height/2); The hematoma clearance rate: preoperative hematoma volume -postoperative hematoma volume/preoperative hematoma volume × 100%. The prognosis was assessed by the modified Rankin scale (mRS) (Table 1) [7].

**Table 1:** Modified Rankin Scale

Score	Patient's condition
0	Complete recovered, no symptoms
1	Despite symptoms, there was no significant dysfunction
2	Mild disability; Can live independently, but part of daily life can be restored
3	Moderate disability; Unable to live independently, but walking with crutches
4	Between moderate and severe disability; Unable to walking independently
5	Severe disability; Incontinence of urine and urination, completely dependent on others in daily life

Self care: 0–2; Disability: 3–5

### Informed consent

The institutional review board at Xi'an Daxing Hospital and Xi’an Fengcheng Hospital approved the study, which was granted a waiver for patients (or their guardians) consent owing to the retrospective nature of the present study. This research conforms with the Declaration of Helsinki. The datasets used and/or analysed during the current study are available from the corresponding author on reasonable request.

### Statistical analysis

Data were analyzed using statistical product and service solutions software version 22.0. Continuous variables were expressed as mean ± standard deviation or median. Differences between variables were evaluated using the independent samples t-test. Categorical variables were expressed as a ratio (%), and differences between variables were compared using the chi-squared test. Data were deemed significant if P < 0.05 for all tests.

## Results

There was no significant difference in age, GCS score at admission, preoperative hematoma volume and postoperative GCS score between the two groups (P > 0.05) (Table 2).

**Table 2:** Comparison of general clinical data between the two groups

	Neuroendoscopic-assisted group (n = 93)	Mini-open craniotomy (n = 91)	P value
Gender			0.266
Male	62	56	
Female	31	35	
Age (years)	55.8 ± 8.7	56.8 ± 7.1	0.394
Admission GCS	8.9 ± 1.2	8.7 ± 0.9	0.203
Preoperative hematoma volume (ml)	48.4 ± 6.1	46.9 ± 7.8	0.147
Operation time (min)	107.9 ± 6.7	108.8 ± 7.8	0.402
Intraoperative blood loss (ml)	87.3 ± 3.6	86.2 ± 4.4	0.064
Postoperative GCS	10.8 ± 0.7	10.6 ± 1.2	0.168
Postoperative hematoma volume (ml)	5.7 ± 3.0	5.1 ± 2.3	0.130

The operation time, blood loss, hematoma clearance rate in the mini-open craniotomy group were more than those in the neuroendoscopic-assisted group, but there was no significant difference between the two groups (P > 0.05). (Table 2).

**Fig. 1:**
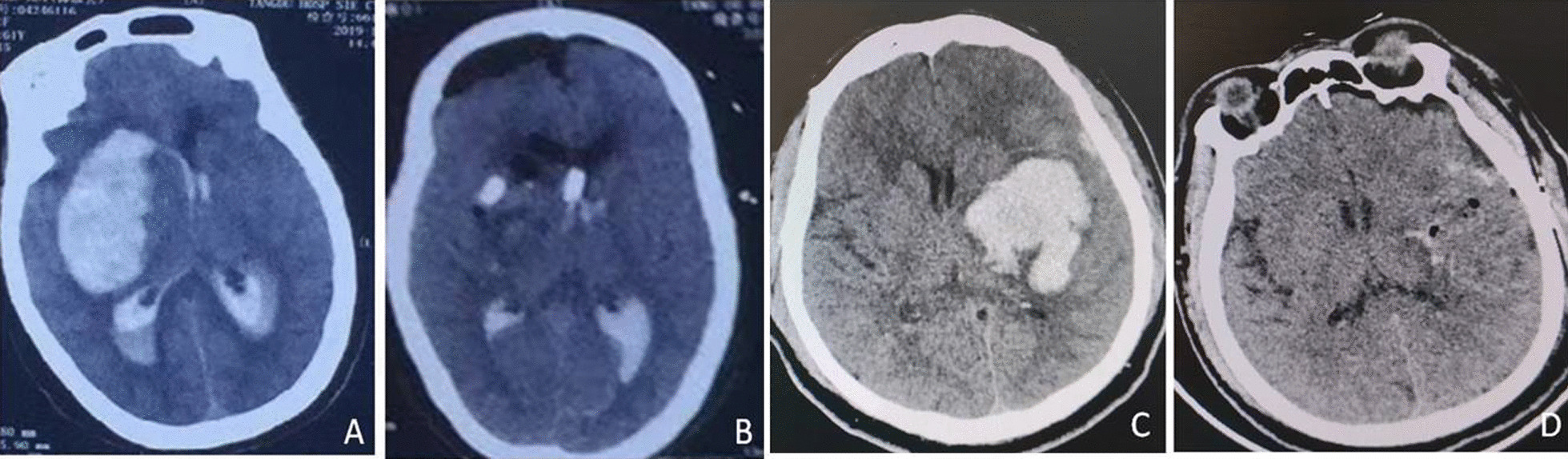
**A** CT images before Neuroendoscopic-assisted surgery; **B** CT images within 24 h after Neuroendoscopic-assisted surgery, Intraventricular hematoma wastreates with extracerebral drainage; **C** CTimages before mini-open caniotomy surgery; **D** CT images within 24 h after mini-open craniotomy

Analysis of the shape of hematoma. (1) spherical hematoma: there was no significant difference in hematoma clearance rate between the mini-open craniotomy group and the neuroendoscopic-assisted group. (2) For rugby hematoma, the hematoma clearance rate of the neuroendoscopic-assisted group was significantly higher than that of the mini-open craniotomy group. The difference was statistically significant (Table 3, Fig. 1).

**Table 3:** Comparison of the clearance rate of hematoma with different shapes between the two groups

Hematoma Shape	Neuroendoscopic-assisted group(%)	Mini-open craniotomy(%)	P value
Spherical hematoma	97.7 ± 7.8	98.5 ± 6.4	0.448
Rugby hematoma	95.1 ± 5.9	89.8 ± 6.2	0.001

Postoperative complications: In the mini-open craniotomy group, 2 cases had rebleeding in the operation area, and 3 cases in the neuroendoscopic-assisted group had rebleeding, and no further surgical treatment was required. The hematoma absorbed spontaneously after conservative treatment. In the mini-open craniotomy group, 4 cases had intracranial infection, while 1 case in the neuroendoscopic-assisted group had intracranial infection, and they were cured after anti-infection treatment. There was no incision infection in the two groups.

After 6 months, the mortality rate of the mini-open craniotomy and the neuroendoscopic-assisted group was 8.6% and 8.8%, and the disability rate was 28.2% and 30.1%, there was no significant difference in mortality and disability rate between the two groups (Table 4).

**Table 4:** Comparison of mRS in two groups 6 months after operation

Score	Neuroendoscopic-assisted group (%)	Mini-open craniotomy (%)
0	10 (11.8)	9 (10.9)
1	23 (27.1)	25 (30.1)
2	28 (32.9)	24 (28.9)
3	13 (15.3)	15 (18.1)
4	8 (9.4)	6 (7.2)
5	3 (3.5)	4 (4.8)

## Discussion

Hypertensive intracerebral hemorrhage (HICH) is one of the serious complications of hypertension [8]. When the volume of intracerebral hemorrhage is large (supratentorial hemorrhage is more than 30 ml), conservative treatment has a poor prognosis, so surgical treatment can be selected. There have been a large number of reports on surgical treatment of HICH in the past, however, no consensus on the optimal procedure has been reached [9, 10]. At present, the commonly used surgical methods include hematoma puncture and drainage; mini-open craniotomy; neuroendoscopic-assisted; large bone flap craniotomy and large bone flap craniotomy + decompressive craniectomy [4, 10, 11]. However, the appropriate surgical method is very important to improve the survival rate, reduce morbidity and mortality, and improve the quality of life after surgery.

The main purpose of surgery is to relieve the compression of hematoma on the surrounding brain tissue as soon as possible, relieve severe intracranial hypertension and cerebral hernia, save the lives of patients, reduce the secondary brain injury caused by hematoma compression as far as possible and improve the quality of life of patients [4, 12]. Compared with traditional craniotomy, minimal invasive surgery (MIS) has the advantages of small trauma, short operation time, fewer complications and rapid recovery, which has been recognized by clinicians [13]. Mini-open craniotomy and neuroendoscopic-assisted are commonly used MIS at present. However, mini-open craniotomy and neuroendoscopic-assisted have their own advantages and disadvantages [14], how to make an effective choice depends on the state of the patient, the type of hematoma and the experience of the surgeon [15].

Mini-open craniotomy hematoma evacuation for HICH has the advantages of the shortest path, the least vascular injury and the greatest reduction of the traction injury to the brain tissue. The cortex is cut about 0.5–1.0 cm, which can effectively protect the small perforating blood vessels and neural structures, protect the functional hemisphere to the maximum extent [16]. neuroendoscopic-assisted surgery has the advantages of small incision, minimally invasive channel, simple operation, no need to stretch brain tissue, so it can reduce nerve injury [17, 18]. Neuroendoscopic imaging is clear, so the operator can obtain a clear surgical field and can observe the deep structure well, which is conducive to the operation of hematoma clearance. However, there are still some deficiencies in neuroendoscopic-assisted surgery, such as limited visual field, limited working pipe, fish eye effect, and some difficulties in hemostasis in case of deep hemorrhage [19, 20].

We are the first to study the effect of hematoma shape and surgical method on hematoma clearance. For spherical hematoma, there was no significant difference in hematoma clearance rate between the mini-open craniotomy group and the neuroendoscopic-assisted group. However, for rugby hematoma, the hematoma clearance rate of the mini-open craniotomy group was significantly lower than that of the neuroendoscopic-assisted group. The main reason is: for rugby hematoma, neuroendoscopic-assisted has a special advantage, the operation sleeve is placed along the long axis of the hematoma, and the sleeve is slowly withdrawn while the hematoma is cleared, to achieve the maximum hematoma clearance rate.

The effect of the time from bleeding to operation on the operation [9]. Through the study, we conclude that for patients with bleeding more than 48 h, compared with neuroendoscopic-assisted surgery, mini-open craniotomy should be used as much as possible. The main reasons are: over 48 h, the hematoma is organized, and it is relatively difficult for the aspirator to remove. Under the neuroendoscopy, due to the limitation of the operation sleeve, the operation space is limited, the hematoma suction is relatively difficult, and the space for block resection is insufficient. Therefore, the operation time will be prolonged, which is not conducive to the postoperative recovery of patients.

HICH is characterized by acute onset and high mortality and disability rates, which seriously threatens the life of a patient, the mortality of this disease, which occurs within 30 days after onset, is 33.3% to 50.6% [6, 13, 15, 17]. How to reduce the disability rate and the mortality rate has been the focus of clinical attention. Tang et al [8]. concluded that compared with traditional craniotomy, MIS can effectively improve the patient's quality of life. Feng et al. [18] believes that endoscope assisted keyhole technique for HICH can effectively improve the prognosis of patients and reduce the disability rate. We found that the mortality rate of mini-open craniotomy and neuroendoscopic-assisted group was 8.6 and 8.8%, respectively, and the disability rate was 28.2 and 30.1%, respectively. The mortality and disability rates were lower than those of traditional craniotomy. The reasons may be as follows: (1) the operation mode and time were selected properly; (2) the patients were younger and had no obvious other basic diseases; the GCS score was relatively high before operation; (3) the number of samples was relatively small, which could not completely represent all HICH patients; (4) this study was a retrospective study, and there might be a deviation in case selection. Hence further randomized research based on a large population and appropriate follow-up durations is required to provide more information about HICH.

## Conclusion

Both mini-open craniotomy and neuroendoscopic-assisted can effectively remove a hematoma, reduce the mortality of patients. its main reason for short operation time, reduced bleeding, and high hematoma clearance rate. Moreover for rugby hematoma, the hematoma clearance rate of neuroendoscopic-assisted is higher than that of mini-open craniotomy. Although the two surgical methods can improve the survival rate of patients, they do not change the prognosis of patients. Therefore, the choice of surgical methods should be adopted based on the patient's clinical manifestations, hematoma volume, hematoma type and the experience of the surgeon.

## Data Availability

The datasets used and/or analysed during the current study are available from the corresponding author on reasonable request.

## Abbreviations

HICH: Hypertensive intracerebral hemorrhage
ICH: Intracranial hemorrhage
CT: Computed tomography
GCS: Glasgow Coma Scale
mRS: Modified Rankin Scale
MIS: Minimal invasive surgery

## References

[1] Van Asch CJ, Luitse MJ, Rinkel GJ, van der Tweel I, Algra A, Klijn CJ (2010). Incidence, case fatality, and functional outcome of intracerebral haemorrhage over time, according to age, sex, and ethnic origin: a systematic review and meta-analysis. Lancet Neurol.

[2] Muengtaweepongsa S, Seamhan B (2013). Predicting mortality rate with ICH score in Thai intracerebral hemorrhage patients. Neurol Asia.

[3] Yang G, Shao G (2016). Clinical effect of minimally invasive intracranial hematoma in treating hypertensive cerebral hemorrhage. Pak J Med Sci.

[4] de Oliveira Manoel AL (2020). Surgery for spontaneous intracerebral hemorrhage. Crit Care.

[5] Zhang FZ, Wang CY, Zhang L (2015). Effects of neuroendoscopy and craniotomy on hypertensive cerebral hemorrhage. Chin J Neurosurg.

[6] Broderick JP, Brott TG, Tomsick T, Barsan W, Spilker J (1990). Ultra-early evaluation of intracerebral hemorrhage. J Neurosurg.

[7] Kwiatkowski TG, Libman RB, Frankel M (1999). Effects of tissue plasminogen activator for acute ischemic stroke at one year. National institute of neurological disorders and stroke recombinant tissue plasminogen activator stroke study group. N Engl J Med.

[8] Tang Y, Yin F, Dengli F (2018). Efficacy and safety of minimal invasive surgery treatment in hypertensive intracerebral hemorrhage: a systematic review and meta-analysis. BMC Neurol.

[9] Chi FL, Lang TC, Sun SJ (2014). Relationship between different surgical methods, hemorrhage position, hemorrhage volume, surgical timing, and treatment outcome of hypertensive intracerebral hemorrhage. World J Emerg Med.

[10] Sun G, Li X, Chen X (2019). Comparison of keyhole endoscopy and craniotomy for the treatment of patients with hypertensive cerebral hemorrhage. Medicine.

[11] Liang KS, Ding J, Yin CB (2017). Clinical study on minimally invasive liquefaction and drainage of intracerebral hematoma in the treatment of hypertensive putamen hemorrhage. Technol Health Care.

[12] Barlas O, Karadereler S, Bahar S (2009). Image-guided keyhole evacuation of spontaneous supratentorial intracerebral hemorrhage. Minim Invasive Neurosurg.

[13] Fam MD, Hanley D, Stadnik A, Zeineddine HA, Girard R, Jesselson M (2017). Surgical performance in minimally invasive surgery plus recombinant tissue plasminogen activator for intracerebral hemorrhage evacuation phase III clinical trial. Neurosurgery.

[14] Dye JA, Dusick JR, Lee DJ (2012). Frontal bur hole through an eyebrow incision for image-guided endoscopic evacuation of spontaneous intracerebral hemorrhage. J Neurosurg.

[15] Xu X, Chen X, Li F, Zheng X, Wang Q, Sun G, Zhang J, Xu B (2018). Effectiveness of endoscopic surgery for supratentorial hypertensive intracerebral hemorrhage: a comparison with craniotomy. J Neurosurg.

[16] Zheng J-S, Yang F, Qing-Sheng Xu (2010). Treatment of hypertensive intracerebral hemorrhage through keyhole transsylvian approach. J Craniofac Surg.

[17] Gushcha AO, Semenov MS, Lepsveridze LT (2015). Experience of endoscopic removal of hypertensive intracerebral hemorrhage. Zh Vopr Neirokhir Im N N Burdenko.

[18] Feng Y, He J, Liu B, Yang L, Wang Y (2016). Endoscope-assisted keyhole technique for hypertensive cerebral hemorrhage in elderly patients: a randomized controlled study in 184 patients. Turk Neurosurg.

[19] Nakano T, Ohkuma H, Ebina K (2003). Neuroendoscopic surgery for intracerebral heamorrhage comparison with traditional the rapies. Minim Invasive Neurosurg.

[20] Feng Y, He J, Liu B, Yang L, Wang Y (2016). Endoscope-Assisted Keyhole technique for hypertensive cerebral hemorrhage in elderly patients :arandomized controlled study in 184 patients. Turk Neurosurg.

